# Changes in exercise adherence during use of an e-health application in individuals with knee osteoarthritis

**DOI:** 10.3389/fresc.2026.1900991

**Published:** 2026-09-21

**Authors:** Mathilde Pelletier-Visa, Louise Jeannot, Bruno Pereira, Emmanuel Coudeyre, Charlotte Lanhers

**Affiliations:** 1 Service de Médecine Physique et de Réadaptation, Centre Hospitalier Universitaire de Clermont-Ferrand, INRAE, UNH, Clermont-Ferrand, France; 2 Direction de la Recherche Clinique et de L’Innovation, Secteur Biométrie et Médico-Economie, CHU Clermont-Ferrand, Clermont-Ferrand, France

**Keywords:** adherence, function, knee osteoarthritis, mobile applications, pain, physical activity, smartphones

## Abstract

**Context:**

Physical activity and rehabilitation comprise the main elements of the non-pharmacological management of knee osteoarthritis (KOA). Compliance with home-based muscle-strengthening exercises is weak, thus diminishing the beneficial effects of the care process.

**Aim:**

We aimed to explore changes in exercise adherence during the use of the ARTH-e application in individuals with KOA.

**Materials and methods:**

We conducted a prospective observational, open labelled, single-center study.

**Results:**

Of the 32 people with KOA, 26 completed the study (62.5% women). Most had unilateral femorotibial compartment involvement (Kellgren & Lawrence stage II or III) and led a sedentary lifestyle. The Exercice Adherence Rating Scale score decreased between 3 weeks and 3 months (effect size −0.41), with 69% of patients considered non-responders. The Knee injury and Osteoarthritis Outcome Score score decreased (effect size −0.62), while no significant changes were observed in pain measured by Numeric Rating Scale.

**Conclusion:**

Over the three-month follow-up, exercise adherence decreased, no significant changes were observed in pain, and functional scores changed over time. Given the observational design and absence of a control group, no conclusions can be drawn regarding the effectiveness of the application.

## 1 Introduction

Knee osteoarthritis (KOA) is a form of osteoarthritis that affects the knee, leading to the progressive degradation of articular cartilage (1). Although it is common in older adults (2), factors such as obesity (3), previous joint injury (4) or abnormal joint alignment can accelerate its onset (5). The main consequences of this condition include joint pain, knee stiffness and reduced mobility. Ultimately, these symptoms can impair quality of life by limiting daily activities and increasing the risk of loss of independence. In advanced cases, joint replacement surgery may be required (6).

Management of KOA includes both non-pharmacological and pharmacological treatments. Current international guidelines prioritize non-pharmacological strategies, including physical activity (PA), self-management, and weight loss (2) when appropriate. These guidelines are particularly important given the low level of PA performed by people with KOA due to the development of kinesiophobia linked to pain (7). Today, connected objects have taken a predominant place in our daily life, including among older people (8), and may facilitate self-management and support long-term exercise adherence to prescribed exercise programs. Digital interventions can provide personalized and adaptive support that complements conventional rehabilitation (9). However, adherence to home-based exercise tends to decline over time (10, 11), highlighting the need for strategies to sustain long-term engagement. Among these strategies mHealth have emerged as promising tools to support motivation and promote exercice participation (12–14).

Although several mHealth interventions for KOA have been developed internationally, most have primarily evaluated pain and functional outcomes, whereas evidence regarding their association with exercise adherence remains limited and inconsistent. Furthermore, few tools have been designed using a patient-centered approach that incorporates users’ perceived barriers and facilitators. To our knowledge, at the time of study conception, no French-language mHealth application dedicated to KOA self-management had been developed and evaluated. Therefore, there remains a need for digital rehabilitation tools tailored to patients’ needs and specifically designed to support adherence to home-based exercise, a key determinant of long-term outcomes in KOA.

To address this gap, we previously conducted a qualitative study involving individuals with KOA and physicians to identify perceived barriers and facilitators to the use of an mHealth for people with KOA (NCT04120727). Participants highlighted the need for guidance and guidance to perform PA safely because of their fear of worsening their symptoms. Interestingly, no age-specific barriers to the use of digital tools were identified among older participants. Based on these findings, we developed ARTH-e, a patient-centred mHealth application designed for smartphones and tablets. The application includes progressive self-exercise programmes, self-monitoring of pain and exercise progression, and educational content to support self-management. By integrating patient-identified needs into its design and targeting adherence to home-based exercise, ARTH-e aims to address an important gap in current digital rehabilitation strategies for KOA.

The main objective of the ARTH-e 2 study was to explore changes in exercise adherence using the Exercise Adherence Rating Scale questionnaire (EARS) (15), during the use of the ARTH-e application in individuals with KOA. The secondary objectives of the study were to describe changes observed in knee function, assessed using Knee injury and Osteoarthritis Outcome Score questionnaire (KOOS) and pain intensity, assessed using the Numeric Rating Scale (NRS) during the study period.

## 2 Materials and methods

### 2.1 Study design

We conducted a prospective, observational, single-centre pilot study. This exploratory design was intentionally selected as a preliminary step before a future comparative trial. The primary objectives were to describe changes observed during use of the ARTH-e application, assess the feasibility of the intervention and study procedures, and obtain preliminary estimates of outcome variability and effect sizes to inform the design and sample size calculation of a future randomized controlled trial. Consequently, this study was not designed to establish the effectiveness of the intervention or allow causal inference.

The study was approved by the *Sud Est I* ethics committee in September 2020. A summary of the research was submitted by the Sponsor to the French National Agency for the Safety of Medicines and Health Products (Agence Nationale de Sécurité du Médicament et des produits de santé, ANSM) before beginning. The protocol was registered on ClinicalTrials.gov (NCT04750304) on February 10, 2021. Participant recruitment began in January 2022 and ended in July 2022. This study was reported according to the Strengthening the Reporting of Observational Studies in Epidemiology (STROBE) guidelines for observational studies (16).

### 2.2 Participant recruitment and setting

Participants were recruited on a volunteer basis from individuals receiving routine care in the Physical Medicine and Rehabilitation Department (PMR) of the Clermont-Ferrand University Hospital, France, as part of their pathology (routine care) and among healthcare professionals of the same institution (mailing list). Because recruitment was voluntary and conducted in a specialized center, the study population may not be fully representative of the broader population of individuals with KOA. In particular, participants may have had greater digital literacy, higher motivation, and more favorable beliefs regarding exercise and self-management. The general process of the study is shown in Figure 1.

**Figure 1:**
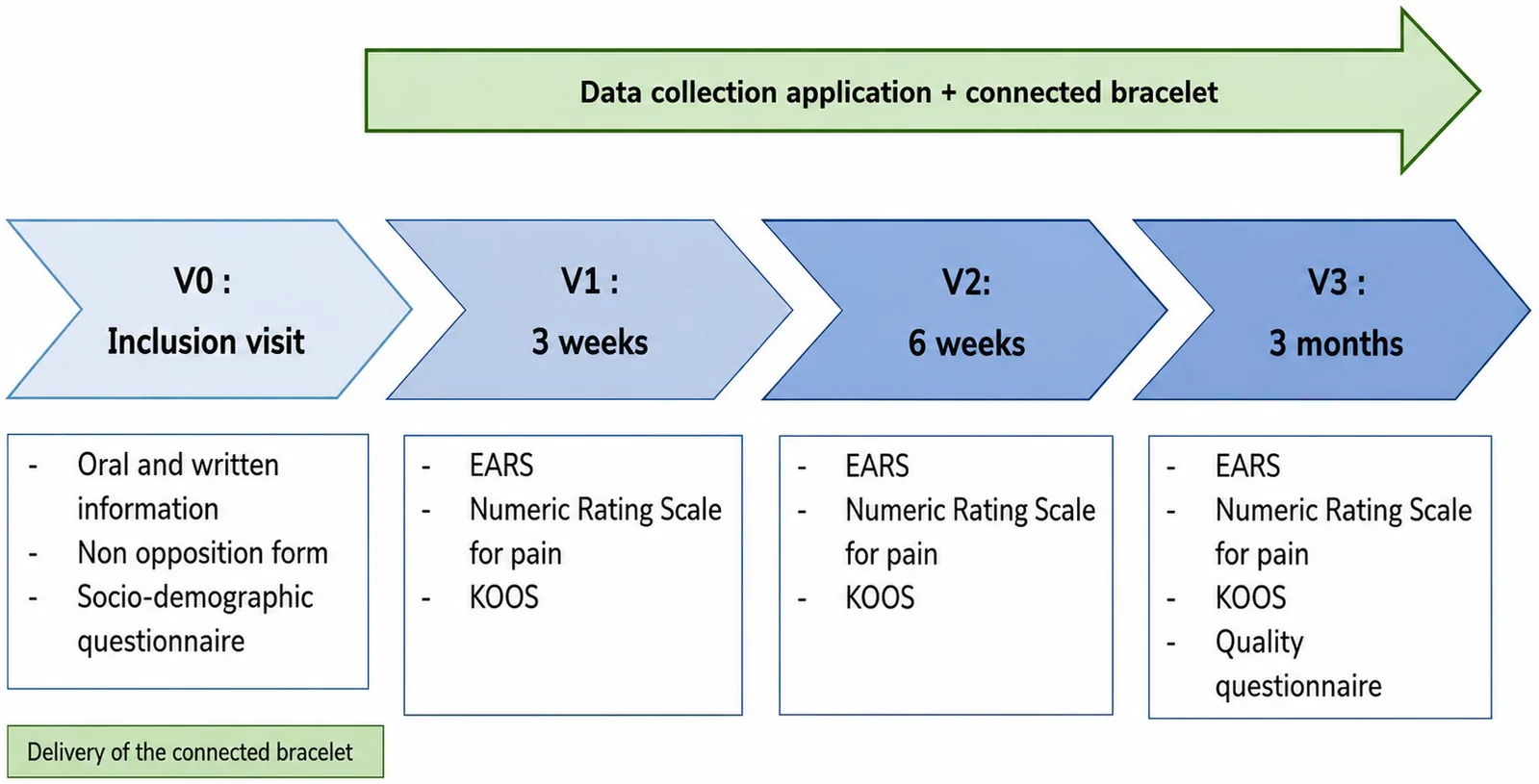
Study design and schedule of assessments during the 3-months follow-up EARS: exercise adherence rating scale; KOOS: knee injury and osteoarthritis outcome score; V: visit.

A Clinical Research Associate (CRA) introduced the study to eligible individuals by phone call. After reflecting, people telephoned the CRA if they decided to participate. Then, an inclusion visit was scheduled with the physician and the CRA, during which a medical examination was performed and the inclusion and non-inclusion criteria were checked. Participants received a copy of the information leaflet and written informed consent was given. Then, the CRA installed the application on the smartphone or tablet with the individual.

### 2.3 Inclusion criteria

We included adults, with no upper age limit, diagnosed with OA in at least one knee according to the American College of Rheumatology (ACR) criteria prior to inclusion by a specialist or non-specialist physician, and symptomatic (17). Participants had their own smartphone or tablet running with at least Android 5 or iOS 11.

Given the exploratory and real-world nature of the study, no additional restrictions were applied regarding symptom duration, baseline pain intensity, radiographic severity, or previous rehabilitation exposure, in order to maximize external validity and reflect routine clinical practice.

### 2.4 Exclusion criteria

We did not include people with comprehension disorders that made interviewing and filling out questionnaires or using the application impossible, people under guardianship, curatorship, or deprived of liberty. During the medical screening, participants with conditions limiting unsupervised exercise (e.g., inflammatory arthritis, neurological disorders, or severe lower-limb musculoskeletal comorbidities) were excluded.

### 2.5 ARTH-e application

The ARTH-e application was developed by the PMR department in collaboration with Openium and is available for download from the Google PlayStore or AppleStore platforms. The interface was designed to ensure simple and intuitive use. The home screen includes a dashboard, 14 predefined exercise programs and randomly suggested programs, as well as a “Did you know?” section providing educational content aimed at improving user’s understanding ok KOA and correcting common misconceptions. The application menu contains six sections: Home, Did you know? My programs, My follow-up, Information and Legal information (Figure 2).

**Figure 2:**
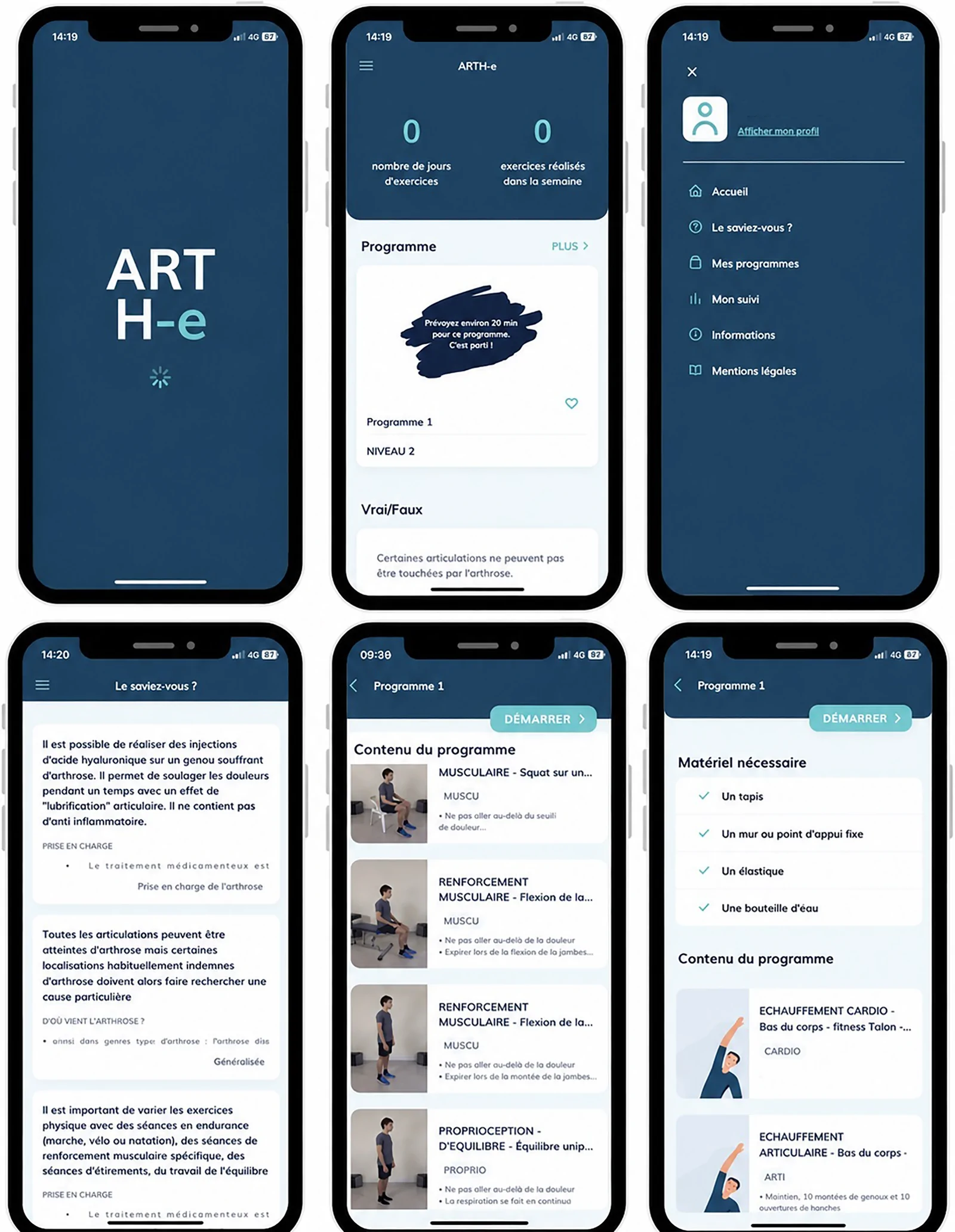
Representative screenshots of the ARTH-E app interface (French version).

Exercise programs are organized into three progressive levels of difficulty and follow a standardized structure composed of five sequential components: cardiovascular warm-up, joint warm-up, muscle strengthening, proprioception and stretching. Exercises primarily target the lower-limb muscles, particularly quadriceps, hamstrings, and hip stabilizers. Content was developed through an interdisciplinary collaboration involving physicians, physiotherapists, and adapted physical activity instructors and in accordance with the French Society of Rheumatology (2019) (6, 18) and the American Academy of Orthopaedic Surgeons (AAOS, 2021) recommendations (19). Once users become familiar with the application they can create personalised sessions by selecting one or more exercises from each of the above- in a guided format (Figure 2).

Each sessions lasts approximately 25 and 30 min and includes short rest periods. Before each session, the application provides required equipment (e.g., mat, chair, ball) and video demonstrations, illustrate correct execution to ensure safety and exercise quality.

During the study, all exercise difficulty levels (beginner, intermediate, and advanced) were available from the outset. The application did not impose predefined progression criteria between levels, allowing participants to freely select the level they considered most appropriate according to their perceived abilities, symptoms, and preferences. No automated progression algorithm is implemented.

Participants were encouraged to perform two to three exercise sessions per week, in accordance with the recommendations provided within by the physician. However, they remained free to choose the timing and content of their sessions throughout the three-month follow-up period. The application does not include automated reminders or push notifications. Engagement relies on voluntary self-management.

Engagement is based on voluntary use and self-management principles rather than behavioural prompting systems.

Personalization is supported through user selection of exercises according to users’s preferences, and perceived needs. Following initial familiarisation, users can construct individualised sessions by combining exercises across the predefined. In addition, pain-related feedback recorded within the application allows users to adapt exercise selection over time according to their tolerance.

Adherence is monitored through self-reported usage data integrated into the application, including exercise completion logs and user-confirmed session validation. Complementary engagement indicators, such as frequency of application use and time spent within exercise modules, are also collected to estimate overall adherence.

### 2.6 Objectives

The main objective of the ARTH-e 2 study was to explore changes in exercise adherence during the use of the ARTH-e application in individuals with KOA.

The secondary objectives were to describe changes in knee function and pain (NCT04750304). In view of the difficulties encountered in other research projects in getting to grips with a mobile application (12), the study also aimed to evaluate the application in terms of both content and form (general user experience, user interface, user expectations), in order to improve its ergonomics and thus limit the obstacles to its use.

### 2.7 Outcomes

Self-administered questionnaires were used, and outcomes were collected by phone by the CRA at 3 weeks, 6 weeks and 3 months after the beginning of the intervention.

The main outcome was exercise adherence using the Exercise Adherence Rating Scale (EARS) questionnaire (15), a self-reported questionnaire consisting of six items rated on a 5 point Likert scale ranging from 0 (completely agree) to 4 (completely disagree), higher scores indicating better adherence to prescribed exercises. Originally developed for individuals with low back pain, the questionnaire was translated, cross-culturally adapted in French and adapted for use in individuals with KOA by our research team. In the present study, EARS scores reflected adherence to the exercise programme and recommended frequency proposed by the physician (two to three sessions per week). The French version of the EARS adapted for individuals with KOA has recently undergone a dedicated psychometric validation study, which is currently under peer review. Briefly, the questionnaire demonstrated satisfactory internal consistency (Cronbach’s *α* = 0.77), excellent test-retest reliability (Lin’s concordance coefficient=0.89), acceptable construct validity, satisfactory Rasch model fit, and responsiveness to change (effect size=0.88). Nevertheless, because these results have not yet been published, findings based on the EARS should be considered exploratory. Because the EARS questionnaire measures adherence to a prescribed exercise program, it was not administered at baseline, as participants had not yet initiated the exercise program delivered through the application. Consequently, the study was designed to describe adherence trajectories during the intervention period rather than changes from baseline.

Secondary outcomes were clinical outcomes. Pain intensity was assessed using a 10-point Numerical Rating Scale (NRS), ranging from 0 (no pain) to 10 (worst imaginable pain).

Knee function was assessed using the Knee injury and Osteoarthritis Outcome Score (KOOS) (20) at 3 weeks, 6 weeks and 3 months of use of the application. The KOOS questionnaire is a patient-reported outcome measure comprising five subscales: pain, stiffness, daily life and function, activities and leisure. Each item is rated on a 5-point Likert scale ranging from 0—“completely agree” to 4—“strongly disagree”. Scores are transformed to a 0-100 scale, where 0 represents extreme knee problems and 100 no knee problems.

Indicators of application use were aslo collected (frequency of opening per week, time spent on the app, rating of each exercise). Application usage data were based on logged exercise sessions rather than simple application openings. Each completed session included information on exercise selection, difficulty level, and self-reported pain, allowing estimation of active exercise duration. Objective physical activity data were planned to be collected using the Garm*in vivo*Smart 4 connected wristband, including number of steps, heart rate, movement intensity, heart rate variability, and energy expenditure. Sixteen participants agreed to wear the device. However, technical difficulties related to data synchronisation and data extraction prevented the retrieval of usable objective activity data. Consequently, no Garmin-derived physical activity outcomes were available for analysis and these data were not included in the present study.

### 2.8 Procedure

They subsequently underwent a medical examination, completed a demographic questionnaire and installed the ARTH-e application on their smartphone or tablet. All the participants attended a one-hour self-management session with the department’s Adapted Physical Activity teacher. The session introduced participants to the exercises available in the application and provided guidance on their safe performance. We asked all participants whether they would like to benefit from a connected watch as part of the study, and only 16 patients agreed to take part. The sub-population who agreed to wear a Garm*in vivo*smart 4 connected bracelet for the collection of intrinsic activity data were issued with the bracelet, which allowed collection of the number of daily steps, daily calorie energy expenditure, etc. These participants were allowed to keep the connected bracelet after the study for their personal use. All data were anonymized.

Two phone calls were then scheduled at 3 and 6 weeks. During these remote interviews, participants were asked to complete the EARS and the KOOS; they were also asked to estimate their level of pain using a NRS. The study was closed with a final phone conversation, three months after the inclusion date, during which the participant was asked to complete the EARS and KOOS questionnaires again, and to estimate their pain level on the NRS (Figure 1).

During the last phone call, participants received a link by email to complete a quality questionnaire about the ARTH-e application, which we will not present in this article. The answers to this quality questionnaire enabled us to improve the application.

### 2.9 Statistical analysis

No formal sample size calculation for hypothesis testing was performed, as this was an exploratory pilot study. However, we aimed to include at least 25 participants with complete follow-up, which was considered sufficient to provide preliminary estimates of changes in EARS scores and their variability. For a quantitative paired before–after outcome, assuming an intra-individual correlation coefficient of 0.50, this sample size allows estimation of a moderate within-subject change with acceptable precision and provides effect size estimates to inform the design of a future randomized controlled trial.

Continuous variables were summarized using medians and interquartile ranges. Normality was assessed using the Shapiro–Wilk test. Longitudinal changes were analyzed using linear mixed-effects models with participant included as a random intercept to account for within-subject correlation. Time (3 weeks, 6 weeks and 3 months) was entered as a categorical fixed effect. Model parameters were estimated using restricted maximum likelihood (REML). Model assumptions were assessed by visual inspection of residual and quantile–quantile plots. Mixed-effects models included all available observations, allowing participants with incomplete follow-up to contribute to the analyses under the missing-at-random assumption. Missing data were not imputed. The main longitudinal analyses were performed using mixed-effects models including all available observations, allowing participants with incomplete follow-up to contribute to the estimates under the missing-at-random assumption. A complete-case sensitivity analysis was also conducted among participants who completed the three-month follow-up.

Analyses were performed using Stata software (version 15; StataCorp, College Station, Texas, USA). A two-sided type I error of 5% was considered statistically significant. Comparisons between responders and non-responders were performed for exploratory and descriptive purposes only. For EARS, a threshold of 5 points was used based on previous literature, although this threshold has not been specifically validated in individuals with KOA. For KOOS, an increase of 8-point change was considered as a descriptive threshold, in line with commonly reported minimal clinically important differences for KOOS domains, but interpretation depends on the subscale and scoring direction. For pain, a 2-point decrease on the numeric rating scale was used as a commonly accepted threshold for clinically meaningful improvement. Given the absence of KOA-specific validation of these responder thresholds in the context of this intervention, responder classifications should be considered exploratory and interpreted with caution.

Given the limited sample size, these analyses were not intended to identify predictors of response. Effect sizes were reported using Hedges’ g for continuous variables and Cramer’s V for categorical variables, in addition to *p*-values. The results are expressed using 95% confidence intervals. Thresholds for EARS, KOOS and pain changes were fixed according to statistical distribution and published literature. Pairwise comparisons between the three assessment time points (3 weeks, 6 weeks and 3 months) were adjusted using Sidak’s procedure to control the family-wise type I error rate.

## 3 Results

### 3.1 Description

Between January 31, 2022 and April 7, 2022, 32 individuals were included in the study. Of these, 26 participants completed the three-month follow-up, and 6 persons were lost to follow-up (Figure 3). The baseline characteristics of participants with complete follow-up and those lost to follow-up were overall comparable. Differences observed for the main demographic and clinical variables were generally associated with small effect sizes (Cohen’s *d* ranging from 0.18 to 0.36 for age, BMI, sedentary time and disease duration) and low Cramér’s *V* values for most categorical variables (generally <0.25, e.g., sex *V* = 0.04, radiographic stage *V* = 0.15, laterality *V* = 0.21, lifestyle *V* = 0.23). Two exceptions were observed for educational level (*V* = 0.43) and, more markedly, type of work (*V* = 0.79). However, these findings were based on very small numbers, particularly for type of work, and should therefore be interpreted with caution. Overall, these findings suggest no major imbalance between groups and support the representativeness of the sample retained for the longitudinal analyses.

**Figure 3:**
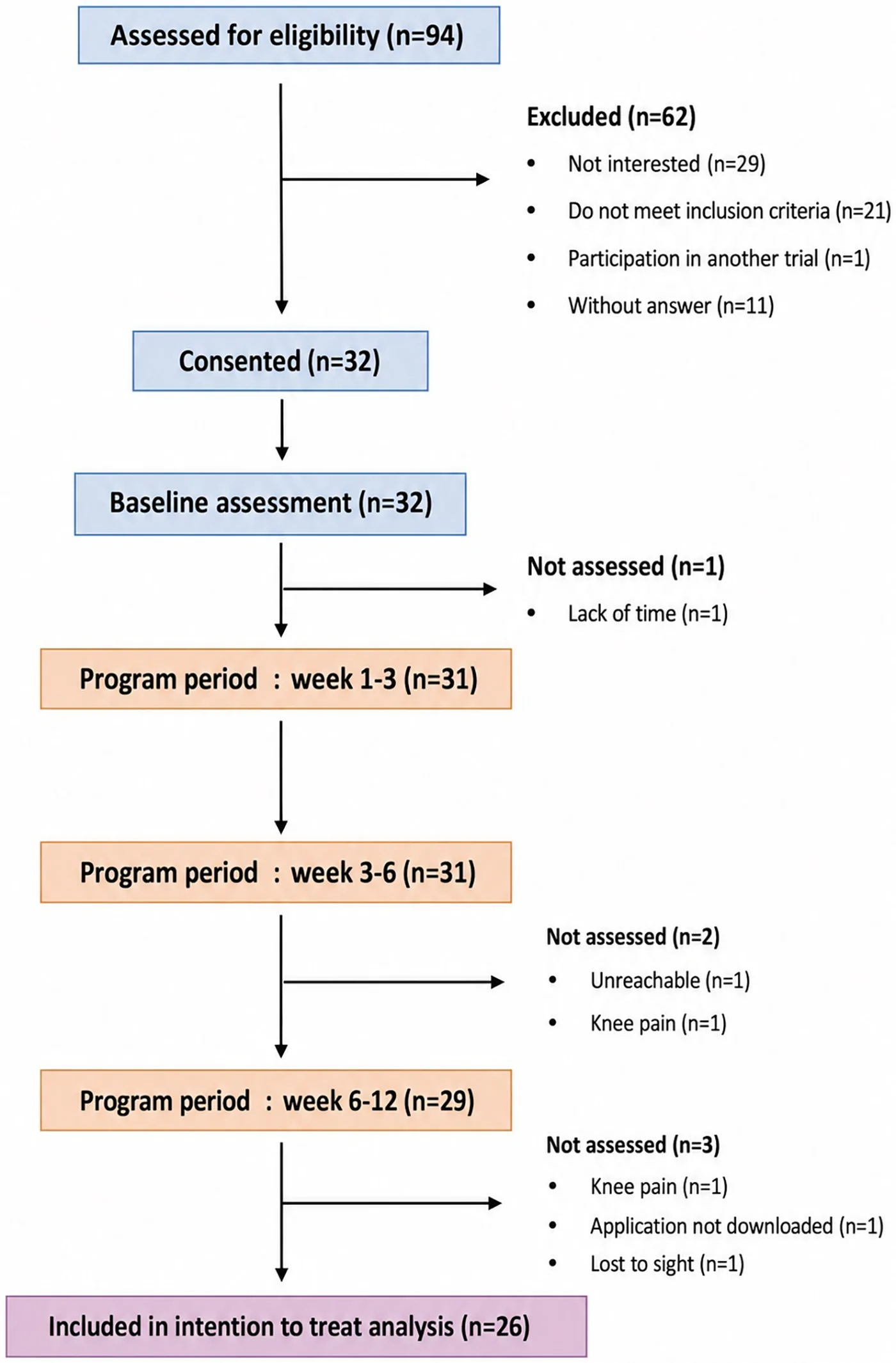
Participant flow diagram.

The median age of the participants was 63.5 [60.0;68.0] years, and 62.5% were women. Half of the participants were retired (*n* = 16, i.e., 50.0% of the 32 patients initially recruited, and *n* = 14, i.e., 53.8% of those who completed the protocol). The majority of working patients had sedentary jobs (respectively *n* = 9, 64.3% and *n* = 8, 72.7%). Most participants had unilateral KOA (62.5%); and 78% had Kellgren & Lawrence grade II or III femorotibial osteoarthritis (21). More details are provided in Table 1.

**Table 1:** Clinical and demographic characteristics of patients with knee osteoarthritis at inclusion.

Patients characteristics	Patients analyzed	
	*n* = 32	*n* = 26
Age, years [median (IQR)]	63.5 [60.0; 68.0]	65.5 [60.0; 68.0]
Sex (F), *n* (%)	20 (62.5%)	16 (61.4%)
BMI, Kg/m² [median (IQR)]	24.11 [21.80; 29.82]	24.16 [22.49; 29.74]
Duration of the evolution of osteoarthritis, months [median (IQR)]	120 [48; 180]	120 [48; 180]
Way of life		
Asset *n* (%)	14 (43.7%)	11 (42.3%)
Sedentary	9 (64.3%)	8 (72.7%)
Physical	2 (14.3%)	0 (0.0%)
Combined	3 (21,4%)	3 (27.3%)
Retired *n* (%)	16 (50.0%)	14 (53.8%)
Invalidity *n* (%)	2 (6.3%)	1 (3.9%)
Radio-clinical damage		
Unilateral (vs. Bilateral) *n* (%)	20 (62.5%)	15 (57.7%)
Internal	23 (71.9%)	17 (65.4%)
External femorotibia	3 (9.4%)	3 (11.5%)
Patellofemoral *n* (%)	13 (40.6%)	11 (42.3%)
Radiological stage (Kellgrenn and Lawrence) *n* (%)		
Stage I	0 (0.0%)	0 (0.0%)
Stage II	14 (43.7%)	12 (50.0%)
Stage III	11 (34.4%)	8 (33.3%)
Stage IV	4 (12.5%)	4 (16.7%)
NC	3 (9.4%)	0 (0.0%)
Associated pathologies		
Diabetes *n* (%)	1 (3.1%)	1 (3.9%)
High blood pressure, *n* (%)	6 (18.8%)	6 (23.1%)
Dyslipidemia *n* (%)	2 (14.3%)	2 (7.7%)
Others *n* (%)	8 (25.0%)	7 (26.9%)
Physical activities	29 (90.6%)	23 (88.46%)
Every day	6 (20.7%)	6 (26.1%)
4–6 times a week	5 (17.2%)	5 (21.7%)
1–3 times a week	17 (58.6%)	11 (47.8%)
Less than once a week	1 (3.4%)	1 (4.4%)
Duration of each physical activity session		
More than 30 min	22 (75.9%)	17 (73.9%)
15–30 min	6 (20.7%)	5 (21.7%)
Less than 15 min	1 (3.4%)	1 (4.4)
Sedentary time, hours [median (IQR)]	5.0 [3.0;8.0]	7.0 [3.5; 8.0]
Osteoarthritis management		
Physiotherapy	22 (68.8%)	17 (65.4%)
Self-exercices programs	3 (9.4%)	3 (11.5%)
Rehabilitation in a specialized center	5 (15.6%)	5 (19.2%)
Spa treatment	10 (31.3%)	9 (34.6%)
Osteopathy	12 (37.5%)	11 (42.3%)
No management	9 (28.1%)	8 (30.8%)
Food supplements	7 (21.9%)	6 (23.1%)
Homeopathy	0 (0.0%)	0 (0.0%)
Tai chi	2 (6.3%)	2 (7.7%)
Acupuncture	4 (12.5%)	3 (11.5%)
None	13 (40.6%)	10 (38.5%)
Technical assistance		
Knee orthesis	5 (15.6%)	3 (11.5%)
Cane	2 (6.3%)	1 (3.9%)
Walker	0 (0.0%)	0 (0.0%)

The median duration of pain was exceeded one year. Overall, 69% of participants had undergone physiotherapy and 37.5% osteopathy. Most participants (75.9%) performed at least 30 min of PA per week, although the majority (58.6%) did not reach the level of PA recommended by the World Health Organization (22). The median daily sedentary time was 5.0 [3.0; 8.0] hours.

### 3.2 Main endpoint

EARS scores are presented in Table 2. In the full cohort, the median EARS decreased from 21.0 [12.0;22.0] at 3 weeks, to 16.0 [13.0;22.0] at 3 months, corresponding to a median change of −1.0 [−6.0; 1.0]. Standardized effect size were −0.37 [−0.73; −0.02], in the full cohort and −0.41 [−0.79; −0.02] in completers.

**Table 2:** Changes in EARS, KOOS and NRS pain scores at 3 weeks, 6 weeks and 3 months. Results were expressed as median [IQR]. “Change from week 3” refers to the variation in scores between the 3-week visit and the follow-up visits at 6 weeks and 3 months. This is calculated for all participants with available data at both time points. “Change from baseline” refers to the change in scores from baseline and is analysed only in participants with complete data at all assessment time points (*n* = 26). EARS and KOOS changes are calculated relative to the 3-week assessment because no baseline EARS measurement was available and the 3-week assessment was considered the first post-intervention evaluation. NRS changes are calculated relative to baseline. EARS, exercise adherence rating scale; KOOS, knee injury and osteoarthritis outcome score; NRS, numeric rating scale.

	Assessments			
Outcomes	Baseline	3 weeks	6 weeks	3 months
All patients				
EARS (*n* = 31, 29, 26)		21.0 [12.0; 22.0]	18.0 [12.0; 22.0]	16.0 [13.0; 22.0]
Change from week 3			−2.0 [−3.0; 2.0], *p* = 0.224	−1.0 [−6.0; 1.0], ***p*** **=** **0.029**
KOOS (*n* = 30, 28, 26)		14.0 [5.0; 20.0]	9.0 [4.0; 14.0]	9.0 [2.0; 12.0]
Change from week 3			−2.0 [−7.0; 1.0], ***p*** **=** **0.044**	−4.0 [−11.0; 0.0], ***p*** **=** **0.001**
NRS Pain (*n* = 32, 31, 29, 26)	3.0 [1.0; 6.5]	3.0 [2.0; 6.0]	3.0 [1.0; 5.0]	4.0 [2.0; 5.0]
		0.0 [−10.0; 20.0],	0.0 [−10.0; 10.0],	0.0 [−30.0; 30.0],
Change from baseline		*p* = 0.125	*p* = 0.391	*p* = 0.545
Complete data (*n* = 26)				
EARS	/	21.5 [16.0; 22.0]	19.5 [16.0; 22.0]	16.0 [13.0; 22.0]
Change from week 3			−1.5 [−3.0;2.0], *p* = 0.333	−1.0 [−6.0;1.0], ***p*** **=** **0.021**
KOOS	/	12.0 [4.0; 20.0]	9.0 [5.0; 13.0]	8.5 [2.0; 12.0]
Change from week 3			−1.5 [−5.0; 1.0], *p* = 0.104	−3.5 [−11.0; 0.0], ***p*** **=** **0.038**
NRS Pain	2.6 [1.0; 7.0]	3.0 [2.0; 6.0]	2.5 [1.0; 5.0]	4.0 [2.0; 5.0]
Change from baseline		0 [−10; 10], *p* = 0.306	0 [−10; 10], *p* = 0.748	0 [−30; 30], *p* = 0.756

Values in bold are statistically significant.

For descriptive purpose, 8 participants (31%) met the predefined criterion for improvement at least 5 points (23) between the 3-week and 3-month assessments.

### 3.3 Secondary endpoint

For the 32 patients initially included, the median KOOS score decreased from 14.0 [5.0;20.0] at 3 weeks, to 9.0 [2.0;12.0] at 3 months, with an effect size of −0.59 [−0.95; −0.24]. Among participants with complete data, the median KOOS score decreased from 12.0 [4.0; 20.0] at 3 weeks, to 8.5 [2.0;12.0] at 3 months, with an effect size of −0.62 [−1.00; −0.23] (Table 2).

In the full cohort, median NRS pain scores increase slightly between inclusion and 3 months (respectively 3.0 [1.0;6.5] and 4.0 [2.0;5.0], with a small effect size (0.11 [−0.24;0.46]). Similarly, among participants with complete data, median pain score changed from 2.6 [1.0; 7.0] to 4.0 [2.0;5.0], with an effect size of 0.06 [−0.32;0.45]. (Table 2).

Table 3 summarizes the responders and non-responders to the various study endpoints. Although the overall median KOOS score declined during follow-up, individual responder analyses identified a subgroup of participants [9 patients (35%)] who achieved the predefined MCID (≥8-point increase). These had a lower Body Mass Index (BMI) (22.9 [20.7; 28.9] vs. 24.8 [23.2; 29.7] kg/m²) and a longer duration of osteoarthritis (126 [54; 270] vs. 72 [28; 180] months) than non-responders. The use of technical assistance was also more frequent among responders (22.2% vs. 5.9%).

**Table 3:** Summary of responders and non-responders to the various study endpoints. BMI, body mass index; NR, non-responders; OA, osteoarthritis. Response (R) was defined according to the Minimal Clinically Important Difference (MCID): an increase of 5 points for the EARS, an increase of 8 points for the KOOS, and a decrease of 2 points for the pain Numeric Rating Scale (NRS).

Patients characteristics	Outcomes					
	EARS		KOOS		Pain NRS	
	R (*n* = 8)	NR (*n* = 18)	R (*n* = 9)	NR (*n* = 17)	R (*n* = 14)	NR (*n* = 12)
Age	63 [58; 67]	66 [60; 69]	60 [59; 67]	66 [61; 68]	61 [59; 67]	67 [64; 69]
Sex (female) n(%)	6 (75.0)	10 (56.0)	6 (66.7)	10 (58.8)	9 (64.3)	7 (58.3)
BMI (Kg/m²)	24.2 [20.9;30.4]	24.2 [23.0;29.7]	22.9 [20.7; 28.9]	24. [23.2; 29.7]	25.1 [22.5; 29.8]	23.8 [22.3; 29.2]
Unilateral n(%)	5 (62.5)	10 (55.6)	5 (55.6)	10 (58.8)	7 (50.0)	8 (66.7)
OA duration (months)	132 [120; 180]	60 [26; 156]	126 [54; 270]	72 [28; 180]	96 [60; 222]	120 [24; 180]
Technical assistance n(%)	2 (25.0)	1 (5.6)	2 (22.2)	1 (5.9)	3 (21.4)	0 (0.0)

With regard to the NRS pain scale, 14 patients (54%) were considered responders (improvement ≥ 2 points). Responders tended to be younger (61 [59; 67] vs. 67 [64; 69] years) than the non-responders, with a slightly shorter duration of osteoarthritis (96 [60; 222] vs. 120 [24; 180] months).

Figure 4 illustrates the overlap between responders according to the three outcome measures (EARS, KOOS and NRS). Only three participants (out of 26) met the responder criteria for all three outcomes, and nine patients did not meet the responder criteria for any outcome.

**Figure 4:**
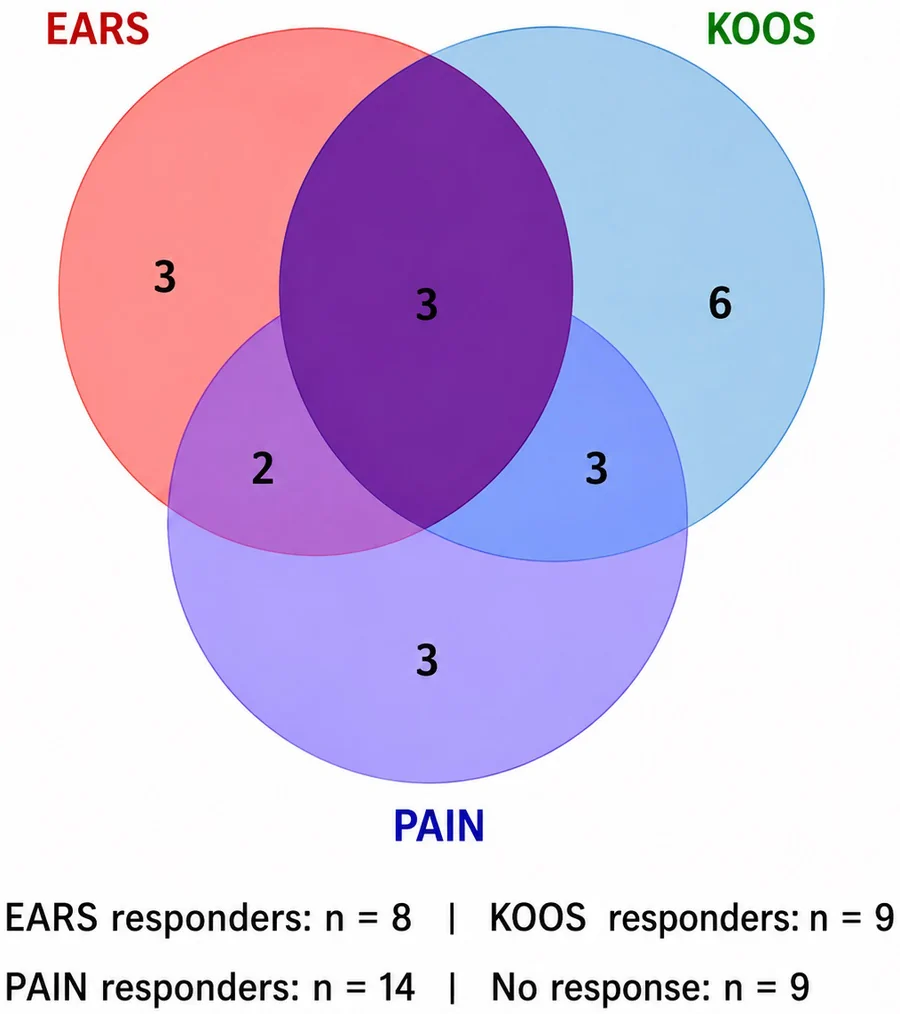
Distribution of participants meeting the response criteria for exercise adherence (EARS), knee function (KOOS), and pain (NRS) using the venn diagram. Overlapping areas represent participants meeting the predefined response criteria for more than one outcome. This exploratory visualization illustrates the heterogeneity of individual response profiles.

Objective physical activity data from the Garmin device could not be analysed because of technical difficulties related to data synchronisation and extraction. Application usage data were successfully collected during the three months of follow-up. Patients used the application for a mean of 48 ± 18 min per opening. Across all participants, level 1 exercise sessions were completed 248 times, level 2 sessions 157 times and level 3 sessions 75 times. Participants could rate each exercise session and the mean rating was 3.7 ± 0.8 on 5.00.

## 4 Discussion

Our study aimed to evaluate changes in exercise adherence associated with the use of the ARTH-e application in individuals with KOA. This pilot study was intentionally designed as an exploratory observational study to provide preliminary estimates of adherence trajectories, variability of outcomes, and effect sizes, as well as to assess the feasibility of the intervention before conducting a fully powered randomized controlled trial. Such preliminary studies are recommended to optimize the design and sample size calculation of subsequent confirmatory trials. Our results showed a significant decrease in the EARS score over the three-month follow-up, with a median change of—5.5 points in the 26 participants with complete data. Given the observational single-arm design, this decline cannot be attributed to the intervention itself and may reflect the well-established tendency for adherence to decrease over time in unsupervised home-based exercise programmes (24). This temporal pattern is therefore more likely to reflect a natural decline in engagement rather than a specific changes associated with the use of the application. Several complementary mechanisms may have contributed to the observed decline in adherence. Exercise adherence in KOA is influenced by a complex interaction of behavioral, motivational, and contextual factors. In particular, engagement decay over time in digital interventions, lack of supervision, perceived exercise difficulty, and absence of structured behaviour change techniques or personalised feedback (25) may have contributed to reduced long-term engagement with the intervention (26, 27). In addition, fluctuating pain (28), fear of symptom exacerbation (29), lack of perceived benefit, and the progressive loss of professional supervision may contribute to disengagement from home-based exercise programmes. Determinants such as self-efficacy, outcome expectations, and self-regulation skills (e.g., goal setting and self-monitoring) have also been shown to play a central role in long-term adherence (30).

Effect sizes for EARS changes were small to moderate (−0.37 to −0.41), suggesting a gradual decline in exercise adherence over time, particularly after the early follow-up period. No significant between-group differences were identified in responder analyses for demographic or clinical variables, although disease duration showed a non-significant trend (*p* = 0.075), warranting further investigation in larger samples (23).

Pain outcomes remained stable over time, with no statistically significant changes observed. Although pain intensity numerically increased from 3.0 to 4.0 in the overall sample, this variation was not statistically significant and does not support any conclusion regarding an improvement in pain symptoms during the study period. In contrast, KOOS scores decreased significantly between 3 weeks and 3 months, indicating a deterioration in self-reported knee function (*p* = 0.038). However, these results should be interpreted cautiously. In the absence of a control group, observed changes may reflect the natural history of KOA, regression to the mean, or the influence of the initial supervised educational session rather than the use of the application alone. This session may have influenced participants’ understanding of osteoarthritis management, confidence in performing exercises independently, and engagement with the rehabilitation programme. However, its specific contribution could not be isolated in the present study. Alternative explanations, including the natural course of KOA, regression to the mean, and the potential influence of the initial face-to-face educational session, may also have contributed to the results. Although the variation between 3 and 6 weeks was not significant, there was a consistent trend towards improved function. Exploratory comparisons between responders and non-responders suggested potential differences according to some demographic and clinical characteristics. Descriptive comparisons suggested that participants classified as responders have a lower BMI and a longer disease duration, while technical aid use was more frequently observed among responders. Similarly, age and disease duration differed descriptively between pain responders and non-responders. However, these observations are based on small subgroup sizes and were not assessed using multivariable modelling. Therefore, they should not be interpreted as predictors or determinants of response but rather as hypothesis-generating signals requiring confirmation in larger studies. The Venn diagram illustrates the great heterogeneity of responses to the intervention according to the three criteria assessed: EARS, KOOS and pain. Only three patients showed a simultaneous positive response on all three dimensions, reflecting the rarity of an overall improvement. The majority of participants showed partial responses, suggesting heterogeneous response patterns across outcome measures. Conversely, nine patients did not respond to any of the criteria, which may reflect limited changes across the evaluated outcomes or a mismatch between the intervention and individual patient characteristics. These results underline the need for a personalized approach that takes into account the diversity of profiles and needs to optimize the impact of rehabilitation interventions. Given the small sample size, these comparisons should be interpreted cautiously and considered hypothesis-generating only, irrespective of statistical significance. Effect sizes were used to describe the magnitude of differences and to help identify variables of potential interest for future studies.

The distribution of responses across outcomes highlights substantial inter-individual variability. Only a small proportion of participants improved across all domains, while others showed isolated or no response, suggesting that digital interventions may produce heterogeneous effects depending on patient profiles and needs.

In addition to the lack of objective physical activity measurements, application usage data provided information on engagement with exercise sessions but did not allow a detailed characterization of adherence behaviour, the absence of more granular metrics (e.g., session frequency per participant, adherence relative to prescribed dose, and variability measures) limits a more precise quantification of adherence behavior. They favored beginner and intermediate exercise levels, which may reflect the perceived difficulty of higher-level sessions or a lack of confidence. This usage behavior emphasizes the importance of adaptability and progressive engagement in digital exercise tools.

Previous studies have reported improvements in pain and functional outcomes with some digital interventions (24–28, 43). More recently, a systematic review and meta-analysis of internet-based telehealth programmes for individuals with hip or KOA reported small to moderate beneficial effects on pain, physical function, quality of life, self-efficacy, and physical activity compared with usual care or minimal interventions (31). These findings support the potential value of digital rehabilitation strategies when implemented within structured care programmes. In contrast, our pilot study did not demonstrate improvements in exercise adherence and showed only limited changes in pain and knee function over the three-month follow-up. Several factors may explain these differences. Unlike many interventions included in the meta-analysis, ARTH-e was evaluated in an exploratory single-arm observational study without a control group and primarily provided autonomous exercise guidance, with limited behavioural support or ongoing professional supervision. By comparison, many successful telehealth interventions combined exercise delivery with personalised coaching, behavioural change techniques, regular clinician feedback, or interactive monitoring (40). These findings suggest that technology alone may be insufficient to overcome the multiple barriers to long-term exercise adherence in individuals with KOA (41). Motivation (32), fluctuating pain (33), self-efficacy, and confidence in performing exercises independently are all recognised determinants of adherence (34, 44). Consequently, future digital interventions should integrate evidence-based behavioural support strategies, including personalised feedback, programme adaptation (35), motivational reinforcement (36), and regular professional follow-up, to optimise long-term engagement (27, 44).

These findings also support the growing interest in hybrid models of care, which combine digital tools with ongoing professional supervision. While e-health applications offer scalable and accessible support for home-based rehabilitation, evidence suggests that their effectiveness may be influenced by integration into structured care pathway involving periodic in-person or remote follow-up by healthcare professionals. Such hybrid approaches may improve motivation, support self-efficacy, and facilitate long-term adherence. This interpretation is consistent with recent evidence suggesting that the effectiveness of digital interventions is enhanced when technology is combined with behavioural support and regular professional follow-up rather than being used as a standalone intervention (31, 42).

Future versions of the application could incorporate behavioural change techniques aimed at supporting long-term adherence, including personalized feedback (37), adaptive goal setting (38), progress tracking, motivational reminders, and symptom-responsive exercise recommendations (27). Furthermore, hybrid models combining digital self-management tools with periodic professional supervision may help maintain motivation, improve self-efficacy, and address barriers to long-term exercise engagement.

### 4.1 Limitations

Several limitations should be acknowledged. First, the observational single-arm design without a control group does not allow causal inference regarding the use of the ARTH-e application, and the observed changes cannot be attributed solely to the intervention. Blinding of participants and investigators was not feasible due to the nature of the digital intervention, which may have introduced expectation or performance biases. In addition, the small sample size (32 participants, including 26 who completed follow-up) limited statistical power and the precision of the estimates. Because the Exercise Adherence Rating Scale (EARS) assesses adherence to prescribed exercises, no baseline adherence measurement was available before initiation of the intervention. Recruitment was conducted on a voluntary among patients from a single specialised rehabilitation centre and among healthcare professionals from the same institution, and required ownership of a compatible smartphone or tablet. Consequently, participants were likely to have greater digital literacy, motivation, and engagement in self-management than the general population of individuals with KOA, limiting the generalisability of the findings. Moreover, all participants received a one-hour face-to-face educational session before using the application. This session may have influenced subsequent adherence, self-efficacy, confidence in performing exercises, and clinical outcomes by providing information about KOA, exercise benefits, and strategies for self-management. However, its specific effect could not be independently assessed, as no data were collected regarding participants’ perceptions of this session or behavioural changes following education. Therefore, the observed findings should be interpreted as reflecting the combined influence of the educational session and the ARTH-e application rather than the application alone. Concomitant treatments, rehabilitation programmes and changes in usual care during follow-up were not controlled and may also have contributed to the observed outcomes. Furthermore, objective physical activity data collected using the Garmin wearable device could not be analysed because of technical issues. Consequently, changes in physical activity could not be objectively quantified or compared with self-reported adherence measures, limiting the ability to determine whether reported exercise engagement accurately reflected actual physical activity behaviour and introducing a risk of reporting bias. Finally, although the French version of the EARS adapted for KOA was developed using established cross-cultural adaptation procedures, its validation study is currently under peer review and its psychometric properties are not yet publicly available. A Hawthorne effect cannot be excluded, as participants may have modified their behaviour because they were aware of being observed. Overall, these limitations highlight the exploratory nature of this pilot study and support the need for adequately powered randomized controlled trials to confirm these preliminary findings.

## 5 Conclusion

In conclusion, this study evaluated changes in symptoms and exercise adherence over a three-month period in individuals with KOA. Adherence to prescribed exercise decreased over time, and no significant improvement in pain was observed. Functional outcomes worsened over time, with a decrease in KOOS function scores, but these changes cannot be attributed to the application. Given the observational design and absence of a control group, these findings should be interpreted cautiously and do not allow conclusions regarding effectiveness. Any observed changes require confirmation in a future adequately powered randomized controlled trial. These findings should be considered hypothesis-generating only. They provide preliminary information on the evolution of exercise adherence during use of the ARTH-e application and will inform the design, sample size calculation, and outcome selection of future randomized controlled trials evaluating its effectiveness. A prospective randomized controlled trial (ARTH-e 3 study), is planned to compare standard care alone with use of the ARTH-e application in addition to standard care (NCT06359171) (39).

## Data Availability

The raw data supporting the conclusions of this article may be made available by the authors upon reasonable request to the corresponding author.
